# Applications of MR Finger printing derived T1 and T2 values in Adult brain: A Systematic review

**DOI:** 10.12688/f1000research.160088.1

**Published:** 2025-01-09

**Authors:** Riyan Mohamed Sajer, Saikiran Pendem, Rajagopal Kadavigere, Priyanka -, Shailesh Nayak S, Kaushik Nayak, Tancia Pires, Obhuli Chandran M, Abhijith S, Varsha Raghu

**Affiliations:** 1 Department of Medical Imaging Technology, Manipal College of Health Professions, Manipal Academy of Higher Education, Manipal, Karnataka, 576104, India; 2 Department of Radio Diagnosis and Imaging, Kasturba Medical College, Manipal Academy of Higher Education, Manipal, Karnataka, 576104, India

**Keywords:** MR Finger printing, Glioma grades, Meningioma, Multiple sclerosis, Brain trauma, IDH mutation

## Abstract

**Introduction:**

Magnetic resonance imaging (MRI) is essential for brain imaging, but conventional methods rely on qualitative contrast, are time-intensive, and prone to variability. Magnetic resonance finger printing (MRF) addresses these limitations by enabling fast, simultaneous mapping of multiple tissue properties like T1, T2. Using dynamic acquisition parameters and a precomputed signal dictionary, MRF provides robust, qualitative maps, improving diagnostic precision and expanding clinical and research applications in brain imaging.

**Methods:**

Database searches were performed through PubMed, Embase, Scopus, Web of science to identify relevant articles focusing on the application of MR finger printing in the adult brain. We utilized the preferred reporting items for systematic reviews and meta-analysis guidelines to extract data from the selected studies.

**Results:**

Nine articles were included in the final review, with a total sample size of 332 participants. In healthy brains, notable regional, sex, age, and hemispheric variations were identified, particularly in the corpus callosum and thalamus. MRF effectively differentiated meningioma subtypes, glioma grades, and IDH mutation status, with T2 values providing particularly predictive for glioma classification. In brain metastases, significant relaxometry differences were noted between normal and lesional tissues. For multiple sclerosis, MRF values correlated with clinical and disability measures, distinguishing relapsing-remitting secondary progressive forms. In traumatic brain injury, longitudinal T1 changes strongly correlated with clinical recovery, surpassing T2 values.

**Conclusions:**

The systematic review highlighted MRD as a groundbreaking technique that enhances neurological diagnosis by simultaneously quantifying T1 and T2 relaxation times. With reduced acquisition times, MRF outperforms conventional MRI in detecting subtle pathologies, distinguishing properties, and providing reliable biomarkers.

## Introduction

MRI is crucial for brain imaging offering high-resolution, non-invasive visualization that supports the diagnosis and monitoring of diverse neurological conditions. ^1^ In adults, MRI is widely used to assess neurodegenerative diseases, brain tumors, stroke and psychiatry disorders, with advanced techniques like functional MRI (fMRI) and diffusion tensor imaging (DTI) allowing for in detail evaluation of brain functional and connectivity. ^2– 8^

Conventional MRI methods have limitations in providing consistent quantitative data, it depends on qualitative image contrast which may vary between the scanner and MR operator. The conventional MRI requires multiple sequences to gather diverse tissue information increasing scan time and motion artifacts- especially in pediatric and uncooperative patients. The lack of quantification limit’s objective evaluation leads to variability in interpretation and potentially limits the utility of MRI in some clinical scenarios. ^9– 12^

To overcome this limitation significant effort has been put into developing quantitative approaches. Quantitative imaging helps to better distinguish the physicians to better distinguish between healthy and pathological tissues. Earlier methods measured one parameter at a time, requiring multiple images with specific acquisition parameters held constant, followed by mathematical modelling to estimate relaxation times like T1 and T2. This process was time-consuming, impractical for clinical use due to long scan times and susceptible to motion artifacts. Recent approaches have been proposed to shorten the acquisition time to provide combined T1 and T2 measurements. However, challenges remain in achieving both speed and accuracy. ^13– 17^

MR finger printing (MRF) is an innovative MRI technique that quantitatively captures multiple tissue properties. MRF transforms qualitative MRI to quantitative MRI. It is designed for fast and simultaneous mapping of multiple tissue properties, such as T1, T2 and proton density. MRF varies acquisition parameters including flip angle and repetition time, in a dynamic and a pseudo manner to generate unique signal evolutions or finger prints for different tissues. A precomputed dictionary of simulated signal patterns, based on anticipated combinations of tissue properties, is then used for pattern recognition. Each voxels acquired signal is compared with a dictionary to find the best match, with the associated properties assigned to the voxel to produce quantitative maps. This method improves scan efficiency, reduces susceptibility artifacts, and offers robust, comprehensive tissue characterization, paving the way for enhanced diagnostic and research applications in clinical imaging. ^18– 21^

There are limited studies which investigated the applications of MRF in adult brain. Hence the aim of the study is to assess the application of MRF and investigate the T1 and T2 map values for adult brain studies.

## Methods

### Design

The literature review was conducted using the guidelines proposed by Preferred Reporting items for Systematic Reviews and Meta-analysis (PRISMA) ^22^ (PRISMA checklist – underlying data).

### Literature search strategy

The literature search was conducted across databases such as Scopus, Web of Science, PubMed, Embase to find the relevant original articles ( Table 1). MesH terms such as “MR Finger printing”, “Magnetic Resonance Finger Printing”, “Adult brain”, “Brain Tumors”, “Multiple sclerosis”,“Meningioma”, “Gliomas”, “T1 and T2 relaxometry using MR Finger printing”, “Brain metastases” “Brain trauma” “Healthy Volunteers” with Boolean operators such as “AND” “OR” was utilized (underlying data). Only English-language research involving adult individuals receiving MRI exams were included in the search parameters.

**Table 1.** Study retrieval method.

Database	Number of studies retrieved	Total
PubMed	20	252
Scopus	121	
Web of Science	103	
Embase	8	

### Selection criteria

Inclusion criteria: The screening process followed the Participant’s Intervention Comparison and Outcome (PICO) methodology ( Table 2). The final review included articles that mentioned T1 and T2 relaxometry values of adult brain using MR finger printing technique.

**Table 2.** Participants intervention comparison and outcome methodology (PICO) for determining study selection criteria.

Characteristics	Criteria
Population	Patients undergoing MRI Brain examinations
Intervention	MRI Finger printing
Comparator	T1 and T2 relaxometry values with conventional MRI techniques
Outcomes	T1 and T2 relaxometry values

MRI- Magnetic Resonance Imaging.

Exclusion criteria: Articles such as case studies and reports, conference abstracts, letters, editorial reviews, meta-analyses, or surveys, animal studies, MR finger printing used for non-neuro examinations, T1 and T2 relaxometry values measured used synthetic MR technique were excluded.

### Data extraction

The extracted date includes author and year, parameters assessed, model and vendor, sample size, design, pathology studied, outcome of the study. Two researchers independently assessed data from each article, and any discrepancies were resolved by a third researcher.

### Quality assessment

The quality of the studies was assessed using the NIH quality assessment tool for cohort and cross-sectional studies developed by the National Heart, Lung, and Blood Institute in 2013. ^23^ This tool assesses studies based on criteria with 14 questions focusing on the key research concepts (Underlying data)

### Results

**Figure 1.**
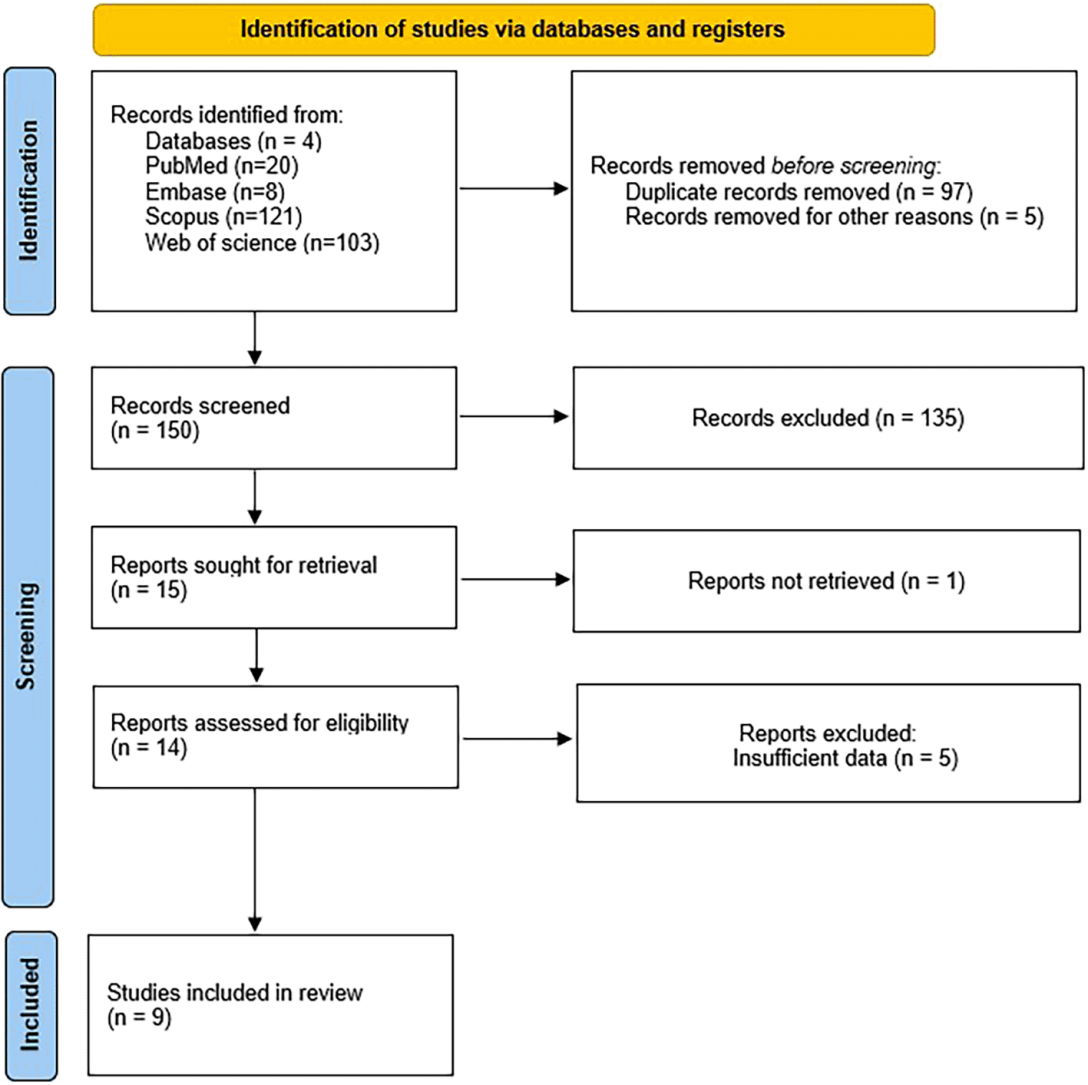
Flow chart for study selection.

A total of nine articles were included in the study ( Figure 1).

### Study selection

The initial search across databases resulted in 252 studies. 97 duplicate entries were removed. The title and abstract of 150 studies were evaluated leading to exclusion of 135 studies as they did not meet the inclusion criteria. A total of 14 articles were retrieved. The full text of 14 articles were assessed for eligibility in total. Five articles were excluded (articles with insufficient data such as not reporting the relaxometry values in detailed table). A total of nine articles were included in the final review.

### Characteristics of selected studies

The review includes studies conducted across three countries, including United states of America (n=6), China (n=2), Austria (n=1). Seven studies have used Siemens Healthcare MRI scanner (Skyra, Verio, Prisma, Trio, 3.0T) and two studies have used GE Healthcare MRI scanner (Discovery 750 W and Discovery 750, 3.0 T). The total sample size across the studies was 332. All nine studies used prospective data collection methods. A summary of the study characteristics and outcomes can be found in Table 3.

**Table 3.** Showing characteristics of selected studies.

Author and Year	Parameters Assessed	Model and Vendor	Sample size	Design	Pathology studied	Outcome of the study
Badve et al. ^24^ 2015	2D MRF (T1 and T2 Relaxometry Maps)	Verio and Skyra 3T MRI (Siemens Healthineers)	56 Healthy volunteers	Prospective	Asymptomatic Volunteers	Age and frontal WM regions were found to be positively correlated, whereas the left substantia nigra and right frontal WM showed negative associations; gender differences were not statistically significant.
Badve et al. ^25^ 2017	MRF (T1, T2 Relaxometry Maps)	Verio, and Magnetom Skyra 3T MRI (Siemens Healthcare)	31 patients	**Prospective**	Brain tumors	Mean T2 values can distinguish between lower grade glioma metastases and solid tumour locations, with solid tumours showing the largest difference. MR fingerprinting for quick, simultaneous T1 and T2 evaluation.
Mostardeiro et al. ^26^ 2021	3D MRF (T1 and T2 Relaxometry Maps)	Discovery MR750 and MR750W 3T (GE Health care)	20 Patients	Prospective	Meningioma	The reliability and validity of these calculations for in vivo, full brain coverage in clinical practice were demonstrated by the study's finding of a linear association between 3D-MRF sequence relaxometry estimations and T1 and T2 relaxation durations in meningiomas.
Zhang et al. ^27^ 2021	MRF T1 and T2 Relaxometry Maps, ADC	Magnetom Skyra scanner 3T MRI (Siemens Healthcare)	46 Patients	Prospective	Meningioma	The study found fibrous and transitional meningiomas had lower T1 and T2 values than meningothelial meningiomas, with the largest AUC for combined T1 and T2 values.
Bai et al. ^28^ 2022	2D MRF, T1 and T2 Relaxometry maps, DWI	Magnetom Skyra 3T MRI (Siemens Healthcare)	51 Patients	**Prospective**	Meningioma	With no discernible variation in T2 signal strength, the study found that soft, moderate, and hard meningiomas differed significantly in T1 and T2 values. Nevertheless, neither the apparent diffusion coefficient value nor the T1 signal intensity showed any discernible changes.
Gerhalter et al. ^29^ 2021	2D radial MRF (T1 and T2 Relaxometry maps), ADC	Magnetom Prisma 3T MRI (Siemens Healthcare)	31 Patients	**Prospective**	Brain Trauma	At timepoint 1, the study's analysis of data from 22 patients and 18 controls revealed no changes in T1, T2, or ADC. Clinical outcome was connected with T1 and T1 changes, with high T1 and serially increasing T1 being most helpful in identifying patients who have not recovered. MRF's T1 was more useful in forecasting three-month results.
Konar et al. ^30^ 2022	T1 and T2 Relaxometry Maps	Discovery MR750W 3T MRI (GE Healthcare)	27 Patients	Prospective	Brain Metastases	BM and normal-appearing brain tissues had significantly different mean T1 and T2 values, according to a pilot study that used Magnetic Resonance Fingerprinting (MRF) to analyze brain metastases. A quick and promising quantitative approach for characterizing BM is the MRF technique.
Springer et al. ^31^ 2022	2D (FISP), T1 and T2 Relaxometry Maps	Magnetom Trio, 3T MRI (Siemens Healthcare)	24 Patients	Prospective	Diffuse Gliomas	According to the study, MRF can improve glioma diagnosis, prognosis, and treatment planning by providing non-invasive information about the genetic composition of gliomas, identifying IDH mutations, distinguishing between glioblastomas and lower-grade gliomas, and monitoring tumor features.
Ontaneda et al. ^32^ 2023	FISP Based MRF, T1 and T2 Relaxometry Maps	Prisma 3 T MRI (Siemens Healthcare)	46 Patients	Prospective	Multiple Sclerosis	Healthy controls, secondary progressive MS, and relapsing remitting MS all had longer T1 and T2 relaxation durations. The thalamus, corpus callosum, and frontal normal-appearing white matter all showed variations, indicating that MRF is a quantitative technique that can be used in clinical settings.

ADC - Apparent Diffusion Coefficient, AUC – Area Under Curve, BM – Brain Metastasis, DWI – Diffusion Weighted Imaging, FISP -Fast Imaging With Steady State Precession, IDH – Isocitrate Dehydrogenase Mutant Gliomas, MRF – Magnetic Resonance Fingerprinting, MS – Multiple Sclerosis,WM – White Matter.

### Quality assessment

**Figure 2.**
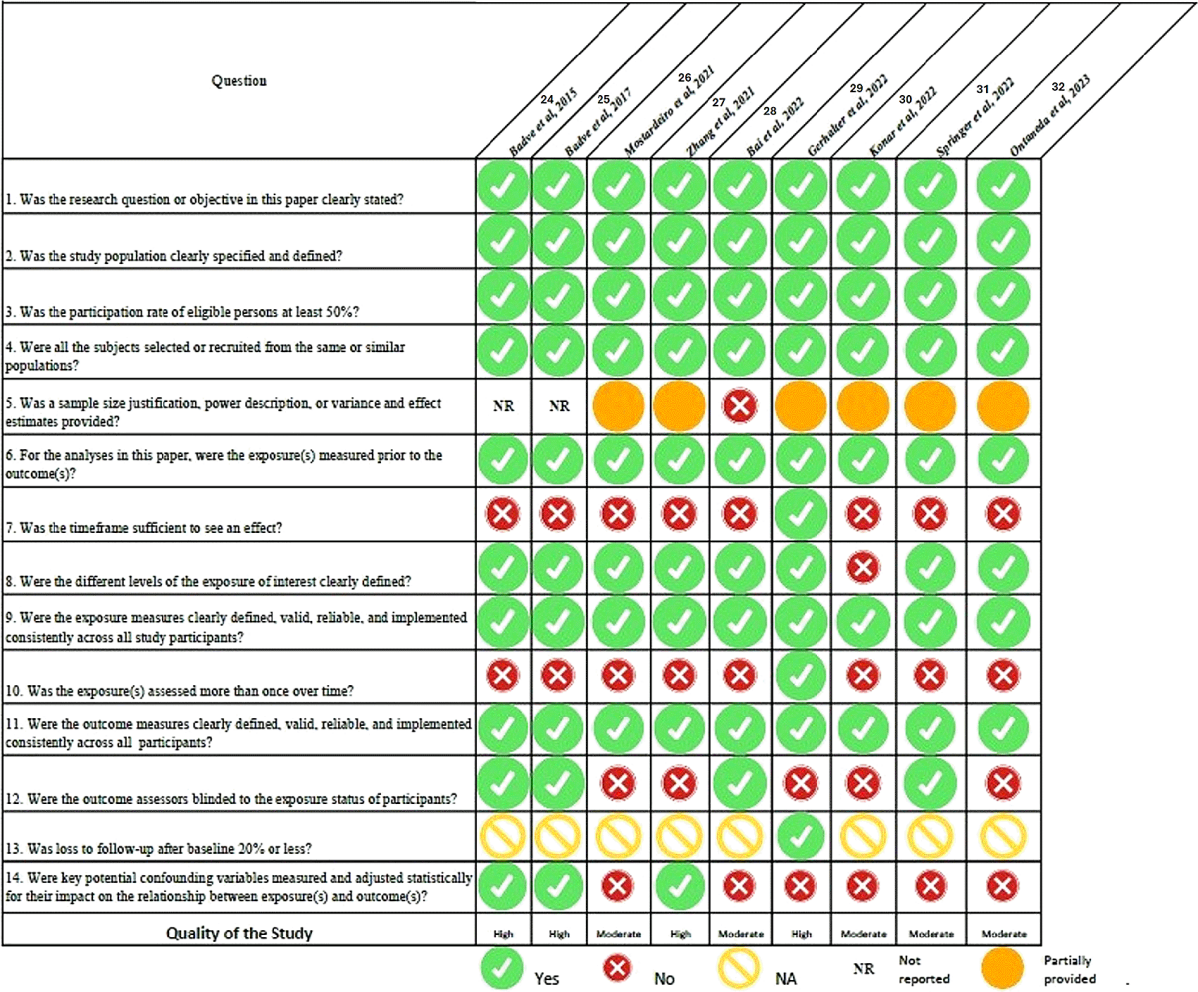
Quality assessment of included articles.

Figure 2 presents a summary of the quality assessment findings. A total of four studies ^24, 25, 27, 29^ was rated as high and five studies ^26, 28, 30, 31, 32^ as moderate quality.

### T1 and T2 relaxometry values in adult brain studies

Summary of “T1 and T2 relaxometry” values for all studies were provided in Table 4.

**Table 4.** Showing the MRF derived T1 and T2 values from included studies.

Author	Pathology/Population	Region/Tumor type/Tumor region	T1 relaxometry values (ms)			T2 relaxometry values (ms)			
			Mean differences between hemispheres			Mean differences between hemispheres			
Badve et al., ^24^ 2015	Healthy volunteers	Superior frontal white matter	-6.29			-1.35			
		Centrum semiovale	-2.98			-1.63			
		Frontal white matter	-13.04			-5.01			
		Caudate nucleus	-4.52			2.78			
		Putamen	-17.00			-1.98			
		Parietal white matter	1.13			-3.92			
		Internal capsule	-14.52			2.99			
		Occipital matter	-17.98			-3.52			
		Temporal white matter	33.02			-0.83			
		Middle cerebellar peduncle	20.08			-1.58			
		Dentate nucleus	-6.44			-4.11			
		Cerebellum	2.72			-3.48			
		Right thalamus (medial-lateral)	91.48			4.55			
		Left thalamus (medial-lateral)	110.91			6.72			
		Corpus callosum (genu-splenium)	-68.00			4.37			
Badve et al., ^25^ 2017	Brain tumors		**GBM (Mean)**	**Metastasis (Mean)**	**LGG (Mean)**		**GBM**	**Metastasis**	**LGG**
		ST T1	1639 ± 247	1324 ± 273	1600 ± 197	ST T2	138 ± 22	105 ± 27	137 ± 37
		PW T1	1578 ± 331	1382 ± 188	1066 ± 218	PW T2	140 ± 27	119 ± 27	127 ± 33
		CWT1	927 ± 133	911 ± 39	873 ± 61	CWT2	69 ± 9	72 ± 6	70 ± 11
Mostardeiro et al., ^26^ 2021	Meningioma		**Mean ±SD**			**Mean ±SD**			
		Meningioma	1429 ± 202			69 ± 27			
		Thalamus	1054 ± 58			27 ± 3			
		Caudate head	1223 ± 52			35 ± 5			
		Centrum semiovale	825 ± 42			29 ± 5			
		Contralateral white matter	799 ± 45			35 ± 4			
Zhang et al., ^27^ 2021	Meningioma		**Mean ±SD**			**Mean ±SD**			
		Meningothelial	661 ± 222			87 ± 40			
		Transistional	491 ± 91			69 ± 15			
		Fibrous meningioma	485 ± 78			65 ± 9			
Bai et al., ^28^ 2022	Meningioma		**Mean ±SD**			**Mean ±SD**			
		Soft	1771 ± 296			108 ± 48			
		Moderate	1549 ± 140			71 ± 14			
		Hard	1442 ± 113			58 ± 13			

Author	Pathology/Population	Region/Tumor type/Tumor region	T1 relaxometry values (ms)				T2 relaxometry values (ms)			
			mTBI (time point 1)		mTBI (time point 2)		mTBI (time point 1)		mTBI (time point 2)	
Gerhalter et al., ^29^ 2022	Brain Trauma	Caudate	12.77 ± 0.56		12.42 ± 0.76		52.27 ± 3.14		51.55 ± 3.45	
		CC body	9.34 ± 0.38		9.11 ± 0.44		40.26 ± 2.9		40.47 ± 2.56	
		CC genu	8.54 ± 0.49		8.31 ± 0.41		36.73 ± 3.1		36.08 ± 2.18	
		CC splenium	8.44 ± 0.35		8.39 ± 0.4		38.53 ± 2.04		39.25 ± 2.46	
		CR	0.45 ± 0.03		0.44 ± 0.02		39.86 ± 1.92		40.42 ± 2.07	
		FWM	8.25 ± 0.3		8.26 ± 0.3		36.77 ± 2.57		37.34 ± 2.54	
		Pallidus	9.17 ± 0.37		9.35 ± 0.87		34.03 ± 2.09		35.91 ± 5.15	
		Putamen	10.71 ± 0.47		10.85 ± 0.33		43.12 ± 2.38		44.3 ± 2.65	
		PWM	8.4 ± 0.25		8.45 ± 0.24		40.54 ± 1.96		41.48 ± 2.41	
		Thalamus	11.09 ± 0.4		10.97 ± 0.29		44.74 ± 2.07		45.14 ± 1.4	
		cGM	13.77 ± 0.29		13.63 ± 0.39		54.5 ± 2.6		54.35 ± 2.81	
		gWM	9.84 ± 0.31		9.83 ± 0.42		42.03 ± 1.84		42.6 ± 2.35	
Konar et al., ^30^ 2022	Brain metastases		**Mean ±SD**				**Mean ±SD**			
		White matter	840 ± 42				78 ± 4			
		Gray matter	1205 ± 31				108 ± 11			
		Cerebrospinal fluid	4233 ± 406				442 ± 56			
		Untreated BM	2035 ± 278				168 ± 47			
		Treated BM	2163 ± 258				141 ± 40			
Springer et al. ^31^ 2022	Diffuse gliomas		**Mean ±SD**				**Mean ±SD**			
		Solid part	0.913				0.775			
		Edema < 1 cm	0.882				0.884			
		Edema >1 cm	0.983				0.983			
Ontaneda et al. ^32^ 2023	Multiple sclerosis		** Hc**	** MS**	** RRMS**	** SPMS**	** HC**	** MS**	** RRMS**	**SPMS**
		FNAWM	800 ± 39.9	850 ± 69.3	816 ± 37.0	896 ± 76.6	59 ± 5.5	66 ± 9.7	63 ±7.5	70 ± 10.9
		Corpus callosum	818 ± 50.9	968 ± 180.7	914 ± 169.2	896 ± 176.0	52 ±5.8	68 ±24.2	62 ± 19.6	77 ± 27.4
		Caudate	1150 ± 51.7	1190 ± 70.3	1170 ± 49.7	1218 ± 84.7	66 ±10.1	71±11.9	69 ± 10.6	74 ± 13.4
		Thalamus	1059 ± 39.2	1096 ± 57.3	1080 ± 52.6	1118 ± 57.2	55 ±6.5	60 ±7.5	58 ± 6.9	63 ±7.3
		Les T2 w	1081 ± 202.3	1180 ± 148.7	1107 ± 118.3	1276 ± 131.3	89 ±34.2	96 ±20.5	87 ±16.1	108 ± 20.0
		Les T1 w	1145	1507 ± 297.7	1408 ± 270.1	1662 ± 296.0	87.5	128 ± 38.2	118 ± 35.0	139 ± 40.1

BM – Brain Metastasis, CC – Corpus Callosum, CR – Corona Radiata, CW – Contralateral White Matter, FNAWM - Frontal Normal Appearing White Matter, FWM – Frontal White Matter, GBM - Glioblastoma Multiforme, gWM – global White Matter, HC – Healthy Control, Les T1w - Lesions On T1-Weigthed Images, Les T2 w - Lesions On T2-Weigthed Images, LGG – Lower Grade Gliomas, mTbi – mild Traumatic Brain Injury, MS - Multiple Sclerosis, ms – millisecond, PWM – Posterior White Matter, PW – Peritumoral White Matter, RRMS - Relapsing Remitting Multiple Sclerosis, SD – Standard Deviation, SPMS - Secondary Progressive Multiple Sclerosis, ST – Solid Tumor, cGM -, cortical Gray Matter.

### MRF derived T1 and T2 values in healthy brain

A study in healthy volunteer ^24^ identified notable differences in brain tissue relaxometry metrics, particularly in the corpus callosum (CC) and thalamus. Within the CC, the splenium showed significantly higher T1 values but lower T2 values compared to the genu (p<0.0001). In thalamus, the medial components exhibited higher T1 and T2 values than the lateral components (p<0.001). T1 relaxometry showed significant increase with age observed in the genu of the CC, in three frontal white matter (WM) regions (p<0.05) and decrease trends were identified in the left substantia nigra (SN) (p=0.0004; R ^2^=0.24) T2 showed significant increase with age in the left frontal WM and the medial left thalamus (p<0.05) and decrease trends were identified in the bilateral SN. Men showed significantly higher T1 values in the left temporal WM, bilateral cerebellar hemispheres, and pons compared to women (p<0.05). For T2 relaxometry, a significant difference was found in the right lentiform nucleus between sexes (p<0.05). Data from right-handed individuals revealed differences between hemispheres. Significant asymmetries were noted in frontal WM (p=0.02), parietal WM (p<0.0001) and other regions.

### MRF derived T1 and T2 values in meningioma

Three studies evaluated the usefulness of MRF in meningioma classification. A study by Mostarderio et al. ^26^ revealed that meningiomas had significantly longer T1 relaxation times compared to the thalamus (p=0.001), corpus striatum (CS; p<0.001), and cerebral white matter (CWM; p<0.001). Similarly, T2 relaxation times of meningiomas were significantly longer than those of the thalamus, CS, and CWM (p<0.001). Pairwise comparison showed that deep gray matter structures such as the thalamus and corpus striatum, had longer relaxation times than white matter (p<0.001). Another study by Zhang et al. ^27^ studied MRF-derived T1 and T2 relaxation times to distinguish meningioma subtypes and compared them to normal brain tissue. Meningothelial meningiomas had significantly higher T1 and T2 values than translational (p=0.002) and fibrous (p=0.002) for T1, p=0.001 for T2) subtypes. No significant differences were found between transitional and fibrous meningiomas (p=0.936 for T1, p=0.617 for T2). The ROC analysis demonstrated the diagnostic efficacy of T1 and T2 relaxation times in differentiating meningioma subtypes. For distinguishing meningothelial and transitional meningiomas, the AUCS were 0.819 for T1, 0.822 for T2, and 0.826 for the combined T1 and T2 values, with optimal thresholds of 1564 ms (T1) and 72 ms (T2), both achieving 80% sensitivity and 83.3 specificity. In differentiating meningothelial from fibrous meningiomas, the AUCs were higher;0.841 for T1, 0.874 for T2, and 0.903 for the combined T1 and T2 values. The optimal thresholds were 1563 ms for T1 and 71 ms for T2, with T2 providing a sensitivity of 86.67% and specificity 84.62%, while the combined values achieved a sensitivity of 80% and specificity of 92.31%.

Study by Bai et al. ^28^ reported that MRF derived T1 and T2 values showed significant differences between the three consistencies (p<0.05), with combined T1 and T2 values achieving the highest diagnostic performance (AUC up to 0.994 for soft vs hard meningiomas) and 100% sensitivity and specificity. T2-weighted signal intensities distinguished soft meningiomas from moderate and hard subtypes (p<0.05) but could not differentiate moderate from hard ones. ADC values and T1-weighted signal intensities were not significantly different across consistencies (p=0.085 and p>0.05, respectively). A multi-class model combining T1, T2 and ADC values achieved 100% prediction efficiency for all subtypes.

### MRF derived T1 and T2 values in gliomas

A study by Badve et al. ^25^ 2017 reported MRF-derived T1 and T2 relaxometry to differentiate glioblastomas (GBMs), lower-grade gliomas (LGGs), and metastases (METs). Patients with LGGs were younger than those with GBMs or METs, with no significant differences in steroid usage or contralateral white matter (CW) metrics. Mean and standard deviation (SD) of T1 and T2 values in solid/enhancing tumor regions (ST) regions were significantly difference from CW across all types of tumor types (p<0.0001). Significant differences were also observed in peritumoral white matter (PW) between GBMs and METs, with mean T1 remaining significant. LGGs and METs differed significantly in ST and PW parameters, with mean T2 in ST regions remaining significant (p<0.05). IDH1 mutation was present in 4 of 6 LGGs but absent in all GBMS. ROC analysis identified the mean T2 of ST regions as the best single predictor for distinguishing GBMs from METs (AUC=0.86, p<0.0001).

Springer et al. ^31^ reported MRF derived T1 and T2 relaxometry values, ADC values, and rCBV values across IDH-mutant and IDH-wildtype gliomas, as well as between tumor components:solid tumor, peritumoral edema, and normal-appearing white matter (NAWM). T1 and T2 values, along with ADC values, were significantly higher in IDH-mutant gliomas compared to IDH-wildtype gliomas in the solid tumor region (p=0.024, p=0.041, and p<0.001, respectively), while rCBV values were higher in IDH-wildtype gliomas but not statistically significant (p=0.252). In perilesional edema, IDH-wild type gliomas exhibited significantly higher T1 and T2 values than IDH mutants (p=0.038 and p=0.010), but differences in ADC values were not significant (p=0.409).

ROC analysis demonstrated that mean T2 values (AUC=0.754) and ADC values (AUC=0.875) were effective for distinguishing IDH-mutant gliomas without 1p/19q codeletion from IDH-wildtype gliomas. Combining T1 and T2 values improved classification, with sensitivity and specificity reaching 0.773 and 0.321 for T1 thresholds (>1670.9) and 0.727 and 0.286 for T2 thresholds (>84.55). Additionally, MRF T1 and T2 values were significantly higher in LGGs compared to HGGs (p=0.017, p=0.002) and were strongly correlated with conventionally measured T1 and T2 values (r=0.913 and r=0.775, p<0.001). rCBV was significantly higher in HGGs than LGGs (p<0.001).

### MRF derived T1 and T2 values in metastases

A study by Konar et al. ^30^ observed that in normal-appearing tissues, mean T1 and T2 values were gray matter (GM) 1205 ms and 108 ms, white matter (WM) 840 ms and 78 ms, and cerebrospinal fluid (CSF) 4233 ms and 442 ms. Significant differences in T1 and T2 values were observed between normal WM and brain metastases (BM) lesions (p<0.05). untreated BM lesions had mean T1 and T2 values of 2035 ms and 168 ms, while treated lesions had mean values of 2163 ms and 141 ms. However, the differences between treated and untreated lesions were not statistically significant (p>0.05). Heat maps revealed heterogeneity in T1 and T2 values among BM lesions, with T1 values more generally consistent across patients than T2. Slightly lower T1 values were observed in untreated BMs and in one patient with a history of primary lung cancer who had undergone stereotactic radiosurgery (SRS).

### MRF derived T1 and T2 values in multiple sclerosis

Ontaneda et al. ^32^ studied demographic, clinical, conventional, MRI and magnetic resonance finger printing (MRF) characteristics among healthy controls (HC), relapsing-remitting multiple sclerosis (RRMS), and second progressive multiple sclerosis (SPMS) patients. Significant differences were observed between MS and HC across all clinical and MRI measures. RRMS and SPMS groups differed in disease duration, Expanded disability status score (EDSS), Timed 25-foot walk (T2FSW), Nine-hole peg test (9HPT), thalamic and caudate volumes, but not in Brain parenchymal fraction (BPF), les T2w correlated strongly with clinical measures like EDSS, T2SFW, 9HPT and Paced auditory serial addition test (PASAT), while Magnetization transfer ratio (MTR) showed no consistent decline with disease progression.

### MRF derived T1 and T2 values in brain trauma

Gerhalter et al. ^29^ analyzed twenty-two patients with mild traumatic brain injury (mTBI) using quantitative MRI (qMRI) measures at two time points: 24±10 days (range:8-53) and 90±17 days (range: 82-142) post injury. Clinical outcomes improved significantly over time, with reductions in concussion symptoms measured by the Rivermead Post-concussion questionnaire (RPQ) across all subscales (p<0.05) and higher scores on the Glasgow outcome scale-Extended (GOSE, p=0.005). cognitive improvements were reflected in better performance on Brief Test of Adult Cognition by Telephone (BTACT) subsets, including word list recall (p=0.005) and number series (p=0.005), and over all z-score increase (p=0.001). MRI analyses revealed that T1 and ADC values in the caudate and global cortical gray matter (GM) significantly lower in mTBI patients than controls at time point 2 (p<0.05). Longitudinally T1 values demonstrated six times more significant correlations with clinical outcomes at time point 2 compared to T2 values, with strong associations (r>0.7) between genu T1 and RPQ scores. FA decreased significantly in regions such as the corpus callosum and thalamus, while T2 values in the palladium increased (p<0.05). Predictive analysis revealed that T1 values in the corona radiata, posterior white matter (WM), and splenium at time point 1, and their longitudinal changes, were strong predictors of recovery (AUC >0.80. These results emphasize the importance of T1 relaxation times as robust markers for mTBI progression and outcome prediction.

## Discussion

MRF represents a transformative advancement in MRI technology, addressing many limitations of conventional imaging methods. While traditional MRI provides high-resolution anatomical details, it relies on qualitative image contrasts that can vary between scanners and operators, introducing subjectivity and limiting reproducibility. ^9– 12, 33^ MRF overcomes these challenges by enabling fast, simultaneous, and quantitative mapping of multiple tissue properties, including T1 and T2 and proton density. In the current study, the utility of MRF-derived T1 and T2 maps in providing quantitative insights into brain tissues and lesions for adult studies were assessed.

For MRF derived T1 and T2 values of brain regions in healthy adults, aging effects on white matter (WM) were significant, with increased T1 and T2 values linked to microstructural changes such as demyelination, gliosis and increase free water content. Frontal and parietal WM showed dynamic age-related changes quadratically, while deep gray matter regions like the substantia nigra (SN) exhibited age-related decreases in T2 due to iron deposition. Sex differences revealed higher relaxation times in men for frontal and parietal WM, reflecting potential neurodegenerative susceptibility, while relaxometry of temporal regions, cerebellum, and pons were influenced by sex dimorphism. Hemispheric asymmetry in relaxation metrics was noted in moto-related regions, and the genu and splenium of the corpus callosum (CC) demonstrated distinct relaxometry profiles tied to microstructural differences. This T1 and T2 normative database serves as a valuable reference for future MRF studies across various disease states. Ongoing efforts aim to enhance in-plane resolution, enable comprehensive 3D coverage, and minimize inhomogeneity artifacts, with the goal of advancing MRF into a more efficient and robust quantitative tool for neuro imaging and broader clinical applications. ^24^

For MRF derived T1 and T2 values in meningioma, Mostarderio et al. ^26^ reported that MRF-based T1 and T2 values of the normal structures of the brain were consistent with previously reported 2D acquisitions and demonstrated strong correlations with reference standards. ^24^ The findings align with prior study by Komiyama et al. ^34^ showing higher T1 and T2 values in meningiomas and other intracranial tumors compared to normal tissues, attributed to increased water content. Zhang et al. ^27^ highlighted the potential of MRF in differentiation WHO grade I meningioma subtypes, specifically transitional and fibrous types, from meningothelial meningiomas, which conventional MRI techniques, including T1WI, T2WI, contrast enhanced T1WI, and ADC values, fail to achieve. MRF generated T1 and T2 values correlate with histological differences, with lower values in transitional and fibrous types due to their dense cellularity and collagen content compared to the higher water content in meningothelial types. This differentiation is clinically significant as transitional and fibrous meningiomas have a higher risk of intraoperative hemorrhage and aggressive behaviors linked to 22q chromosomal abnormalities. ^34^ Another study by Bai et al. ^28^ reported the effectiveness of MRF in predicting meningioma consistency, classifying tumors into soft, moderate and hard categories based on quantitative T1 and T2 values. MRF offers precise measurements reflecting tissue composition such as water, collagen and calcium content. Soft meningiomas with higher water content show elevated T1 and T2 values, while harder tumors rich in collagen and calcium, exhibited lower T2 values. These findings underline MRF’s utility in surgical planning by providing accurate, reproducible information that can aid in neurosurgical instrument selection and improve operative outcomes. They also noticed no significant difference in ADC values among soft, moderate and hard meningiomas, consistent with previous research. ^35– 37^ ADC values, influenced by extracellular water fraction and tumor cellularity, are not effective for predicting meningioma consistency, as soft meningiomas primarily contain intracellular water and benign tumors across consistencies exhibit similar cellularity. Thus, ADC lack discriminatory power in differentiating meningioma consistencies.

A study by Badve et al. ^25^ reported that MRF successfully identified differences in solid tumor regions of LGGs and METs, as well as in the peritumoral regions of glioblastomas (GBMs) and LGGs. It faced challenges in differentiating between solid tumor (ST) regions of LGGs and GBMs, likely due to their shared glial origin and similar cellular characteristics. ^38, 39^ The study revealed that T1 and T2 relaxation times are influenced by factors such as cellularity, water content, and molecular composition. In LGGs, distinct tissue origins contribute to significant differences in relaxometry values compared to METs. The subtle differences in T2 skewness between GBMs and LGGs suggest higher cellular density and microvascular proliferation in GBMs. Conversely, the lack of significant T1 and T2 differences between LGGs and GBMs highlights the challenge of differentiating glioma grades using relaxometry alone. Analysis of peritumoral regions showed that GBMs exhibit infiltrative cells mixed with vasogenic edema, whereas METs are surrounded primarily by vasogenic edema with little histological invasion. This cellular and molecular heterogeneity contributes to measurable relaxometry differences in peritumoral white matter, offering a potential diagnostic marker. ^40– 43^ Another study by springer et al. ^31^ reported that MRF demonstrated significant differences in MRF derived T1 and T2 values were observed between IDH-mutant and IDH-wild type gliomas, particularly in solid tumor regions. IDH-wild type gliomas exhibited lower ADC values and higher MRF T1 and T2 values in peritumoral edema, suggesting increased cellularity and greater infiltration compared to IDH-mutant gliomas. MRF provides quantitative results with higher accuracy, repeatability and efficiency than conventional MRI.

MRF derived T1 and T2 values for characterizing brain metastases (BMs) and differentiating between untreated BMs, treated BMs, and normal-appearing brain tissue. MRF-derived metrics revealed significant differences in T1 and T2 values, highlighting tumor heterogeneity and treatment related changes such as microvascular alterations and radiation necrosis. Untreated BMs exhibited higher T1 and T2 values compared to treated BMs, likely reflecting microvascular alterations and radiation effects such as necrosis or increased iron concentration from blood leakage. The study also identified slight discrepancies between MRF and synthetic MRI (MAGiC) values due to methodological differences, with MRF providing consistent results. ^30^

Ontaneda et al. ^32^ explored the use of magnetic resonance finger printing (MRF) in multiple sclerosis (MS) and demonstrated a stepwise increase in T1 and T2 relaxation times in RRMS and secondary progressive MS (SPMS) compared to healthy controls (HC) in both normal appearing and lesional brain tissue. Significant differences in normalappearing brain regions emphasize the widespread effects of MS, while T1 and T2 relaxation times in focal normal-appearing white matter (FNAWM) and lesional tissue differentiated between RRMS and SPMS, suggesting these regions are particularly informative. By providing normalized, multiparametric imaging, MRF holds promise for distinguishing demyelination from other pathological processes, particularly in cortical lesions, which remain challenging with traditional methods.

Study by Gerhalter et al. ^29^ evaluated the potential of T1 and T2 relaxation times, derived using magnetic resonance finger printing (MRF), to predict clinical outcomes following mild traumatic brain injury (mTBI). Key findings include the predictive strength of T1 relaxation times for global functional (Glasgow outcome scale – extended GOSE), symptomatology (Rivermead post-concussion symptoms Questionnaire- RPQ), and neuropsychological (Brief Test of Adult Cognition by Telephone-BTACT) outcomes surpassing T2 and Diffusion tensor imaging (DTI) metrics like fractional anisotropy and ADC. Higher rates of increasing T1 values were consistently associated with worse clinical outcomes, particularly in white matter regions such as the genu, while T2 performed comparably with DTI metrics without yielding significant predictive associations. The predictive utility of T1 values likely reflects underlying pathologies such as cytoskeletal damage, inflammation, and edema, with higher values potentially indicating progressive neuronal loss.

### Limitations

Firstly, the review primarily focused on applications of MRF in adult, future studies can be done in pediatric population. Secondly, the focus on specific conditions, such as brain tumors and multiple sclerosis, overlooks potential applications in the other neurological and psychiatric disorders. Thirdly, many studies included are pilot investigations with small sample sizes, reducing the generalizability of findings and limiting the exploration of MRFs longitudinal and prognostic capabilities. Additionally, the studies often lack direct correlations with molecular or histopathological data, which constrains the understanding of the biological foundations of MRF derived T1 and T2 values.

## Conclusion

The systematic review on the applications of MRF in the adult brain concludes that MRF is a transformative imaging technique with significant potential to enhance the characterization and diagnosis of various neurological conditions. Its ability to simultaneously quantify T1 and T2 relaxation times with reduced acquisition times offers a promising alternative to conventional MRI techniques, particularly for diseases like brain tumors, multiple sclerosis, and traumatic brain injury. MRF demonstrates strengths in detecting subtle pathological changes, differentiating tissue properties, and providing reproducible quantitative biomarkers.

### Ethics and consent

The study did not involve any human participants, and only systematic review was conducted, hence the written informed consent and institutional ethical committee approval (IEC) was not taken.

## Data Availability

No data associated with this article. Figshare: MRF Systematic Review. https://doi.org/10.6084/m9.figshare.28009427.v2 ^44^ This project contains the following underlying data:
• PRISMA check list • MeSH terms • PRISMA Flow chart • Quality assessment questions Data are available under the terms of the Creative Commons Attribution 4.0 International license (CC-BY 4.0)
