# Supplementary material for: Applications of MR Finger printing derived T1 and T2 values in Adult brain: A Systematic review
Source: F1000Res. 2025 Jan 9;14:54. [Version 1] doi: 10.12688/f1000research.160088.1 (PMC11747303; doi:10.12688/f1000research.160088.1)
Supplement: Peer review history

# Reviewer report: Reviewer response for version 1

**DOI:** 10.5256/f1000research.175906.r358477

**Published:** 2025-01-20

**Authors:** Manna Debnath

**Affiliations:** 1 Charotar University of Science and Technology, Changa, Gujarat, India

**Comments**

1. In the conclusion of the abstract, I noticed that "MRD" has been used, which I believe should be corrected to "MRF." Kindly correct it.
2. The introduction is well-written, highlighting the limitations of conventional MRI and the advantages of Quantitative MRI. It provides a solid foundation for the study and conveys the significance of the topic.
3. The study follows the well-established PRISMA guidelines, which enhances its methodological clarity.
4. In Table 1 (Page 4), you have mentioned that the total number of studies is 252, including those from PubMed, Scopus, Web of Science, and Embase. However, in Figure 1 (Page 6), the total number of studies identified is listed as 256 including Database 4, if so, which Databases? Please clarify.
5. On Page 5, under the "Study Selection" section (2nd line), please mention that 5 studies were removed for other reasons, as indicated in the flowchart (Figure 1).
6. The rest of the Results, Discussion, and Conclusion sections are well-written. They provide a clear and comprehensive interpretation of the findings and study outcomes.

Are the rationale for, and objectives of, the Systematic Review clearly stated?

Yes

Is the statistical analysis and its interpretation appropriate?

Not applicable

If this is a Living Systematic Review, is the ‘living’ method appropriate and is the search schedule clearly defined and justified? (‘Living Systematic Review’ or a variation of this term should be included in the title.)

Yes

Are sufficient details of the methods and analysis provided to allow replication by others?

Yes

Are the conclusions drawn adequately supported by the results presented in the review?

Yes

Reviewer Expertise:

Magnetic Resonance Imaging (MRI)

I confirm that I have read this submission and believe that I have an appropriate level of expertise to confirm that it is of an acceptable scientific standard.

# Reviewer report: Reviewer response for version 1

**DOI:** 10.5256/f1000research.175906.r358478

**Published:** 2025-01-17

**Authors:** Shashi Kumar Shetty

**Affiliations:** 1 K S Hegde Medical Academy, Mangalore,, Karnataka, India

The review emphasizes MR Finger printing as a groundbreaking technique for adult brain imaging, offering shift from qualitative to quantitative MRI.

**Major comments:**

 The use of the PRISMA framework and NIH quality assessment tool ensures a methodologically sound review.

 In abstract the phrase “robust, qualitative maps” should be corrected to “robust, quantitative maps”. In conclusion, “MRD” should be corrected to “MRF”

 Introduction provides an overview of the limitations of conventional MRI techniques and effectively sets the stage for the discussion on MR Fingerprinting (MRF).

 Methodology is well written

 Results section presents a detailed summary of the findings from the included studies, focusing on various applications of MR Fingerprinting (MRF) in the adult brain

 Discussion consolidates the key results from the study, including the diagnostic advantages of MRF in conditions such as gliomas, meningiomas and traumatic brain injuries.

 Conclusion succinctly reiterates the diagnostic and clinical advantages of MRF, particularly in providing quantitative metrics like T1 and T2 values that enhance diagnostic precision and reduce acquisition times.

**Minor comments:**

 In introduction, “Conventional MRI methods have limitations in providing consistent quantitative data, it depends on qualitative image contrast which may vary between the scanner and MR operator” to be corrected as “Conventional MRI methods have limitations in providing consistent quantitative data. They depend on qualitative image contrast, which may vary between the scanner and MR operators”

 Terms like “MR Finger printing” and its abbreviation are used inconsistently. Use the abbreviation after the first mention for consistency.

Are the rationale for, and objectives of, the Systematic Review clearly stated?

Yes

Is the statistical analysis and its interpretation appropriate?

Yes

If this is a Living Systematic Review, is the ‘living’ method appropriate and is the search schedule clearly defined and justified? (‘Living Systematic Review’ or a variation of this term should be included in the title.)

Not applicable

Are sufficient details of the methods and analysis provided to allow replication by others?

Yes

Are the conclusions drawn adequately supported by the results presented in the review?

Yes

Reviewer Expertise:

Radiology

I confirm that I have read this submission and believe that I have an appropriate level of expertise to confirm that it is of an acceptable scientific standard.
